# Effect of different prophylactical doses of ondansetron for the hemodynamic stability in patients undergoing cesarean section: a randomized controlled study

**DOI:** 10.3389/fmed.2025.1495721

**Published:** 2025-04-03

**Authors:** Rui Qin, Xiangzhao Xu, Na Zhao, Yongqiang Shi, Yi Chen, Jinhua Chen, Xinli Ni

**Affiliations:** ^1^Department of Anesthesia and Perioperative Medicine, General Hospital of Ningxia Medical University, Yinchuan, Ningxia, China; ^2^Department of Anesthesiology, The People’s Hospital of Nanchuan, Chongqing, China

**Keywords:** ondansetron, prophylactic, hemodynamic stability, cesarean section, spinal anesthesia

## Abstract

**Background:**

5-hydroxytryptamine 3 (5-HT3) receptor antagonists have been reported to reduce post-spinal anesthesia hypotension, though their efficacy remains controversial. We investigated the effect of prophylactic ondansetron on hemodynamic stability in patients undergoing cesarean section following spinal anesthesia.

**Methods:**

Patients scheduled for elective cesarean section (*n* = 120) were randomly allocated to three groups (NS group, 4 mg group, 8 mg group) of 40: those given 4 mL of normal saline (NS), and those given either 4 mg or 8 mg ondansetron (4 mL) before spinal anesthesia. Patient information, maternal systolic blood pressure stability [median performance error (MDPE), median absolute performance error (MDAPE)], the incidence of post-spinal anesthesia hypotension, norepinephrine doses, other adverse events (severe post-spinal anesthesia hypotension, nausea, vomiting, bradycardia, and hypertension), umbilical artery blood gas values, and infant Apgar scores were all recorded.

**Results:**

The primary outcomes (median performance error, MDPE and median absolute performance error, MDAPE) were significantly different among the three groups. (*p* = 0.001, *p* = 0.002). Compared with the NS group, systolic blood pressure (SBP) was maintained closer to baseline in the 4 mg group (*p* = 0.003, *p* = 0.006), as was the 8 mg group (*p* = 0.011, *p* = 0.006). There was a significant difference in the incidence of post-spinal anesthesia hypotension among the three groups (*p* = 0.002). However, only there was a statistical difference between NS and the 8 mg groups in pairwise comparisons (*p* = 0.001). The doses of norepinephrine, the incidences of other adverse events, umbilical artery blood gas, and Apgar scores were not statistically different between the three groups.

**Conclusion:**

Prophylactic 4 mg or 8 mg ondansetron improved hemodynamic stability after spinal anesthesia in cesarean section; however, only 8 mg reduced post-spinal anesthesia hypotension.

**Clinical trial registration:**

Clinicaltrials.gov, NCT05475873.

## Introduction

Spinal anesthesia is the main anesthetic technique for cesarean section (1). However, the associated sympathetic nerve block extensively may lead to a decrease in maternal systemic vascular resistance, resulting in an increased incidence of post-spinal anesthesia hypotension (as high as 75%) (2). Strategies for the prevention and treatment of post-spinal anesthesia hypotension include left uterine displacement by 15°, binding both lower limbs, crystalloid or colloid fluid pre-loading, co-loading (3), prophylactic and therapeutic vasopressor administration (4, 5), and vasopressor administration combined with fluid loading (6).

Extensive sympathetic block leads to post-spinal anesthesia hypotension and also activates the Bezold–Jarisch reflex (BJR) by hypovolemic stimulation of mechanoreceptors in the ventricular wall. The BJR is activated by reduced right cardiac venous return, leading to vasodilation and bradycardia, further lowering maternal blood pressure (7, 8). Studies have shown that 5-HT3 receptor antagonists can bind to left ventricular receptors to eliminate BJR, reducing the incidence of post-spinal anesthesia hypotension and sinus bradycardia. This maintains hemodynamic stability, reducing the dose of vasoactive drugs required (9).

As a 5-HT3 receptor antagonist, ondansetron is mainly used to prevent and treat nausea and vomiting from various causes. Research has shown that intravenous injection of ondansetron before spinal anesthesia can reduce the incidence of hypotension and bradycardia and the dose of vasoactive drugs required (10). However, there is also evidence that prophylactic ondansetron does not reduce post-spinal anesthesia hypotension (11, 12). Because of these contradictory results, further exploration of the application of ondansetron in the prevention and treatment of post-spinal anesthesia hypotension is needed. The specific dose is also uncertain, which still needs further exploration. We hypothesized that prophylactic ondansetron could further improve the hemodynamic stability after spinal anesthesia in cesarean section. This study compared prophylactic two different doses of ondansetron and a placebo control group before spinal anesthesia in full-term pregnant women undergoing cesarean section, examining the effect on hemodynamic stability.

## Methods

### Study design

This prospective, double-blind, randomized controlled trial was conducted at the General Hospital of Ningxia Medical University, Yinchuan, China with Institutional Review Board approval (Ethics number: KYLL-2023-0052). All subjects participating in the trial provided written informed consent. Before patient enrollment, the trial was registered at clinicaltrials.gov (NCTNCT05475873; principal investigator: Xinli Ni, Yi Chen; date of registration: 12 January 2022; URL: https://clinicaltrials.gov/ct2/show/NCT05475873). The trial was conducted from June 2023 to October 2023 in adherence to the applicable Consolidated Standards of Reporting Trials guidelines and following the Declaration of Helsinki.

### Participants

**Figure 1:**
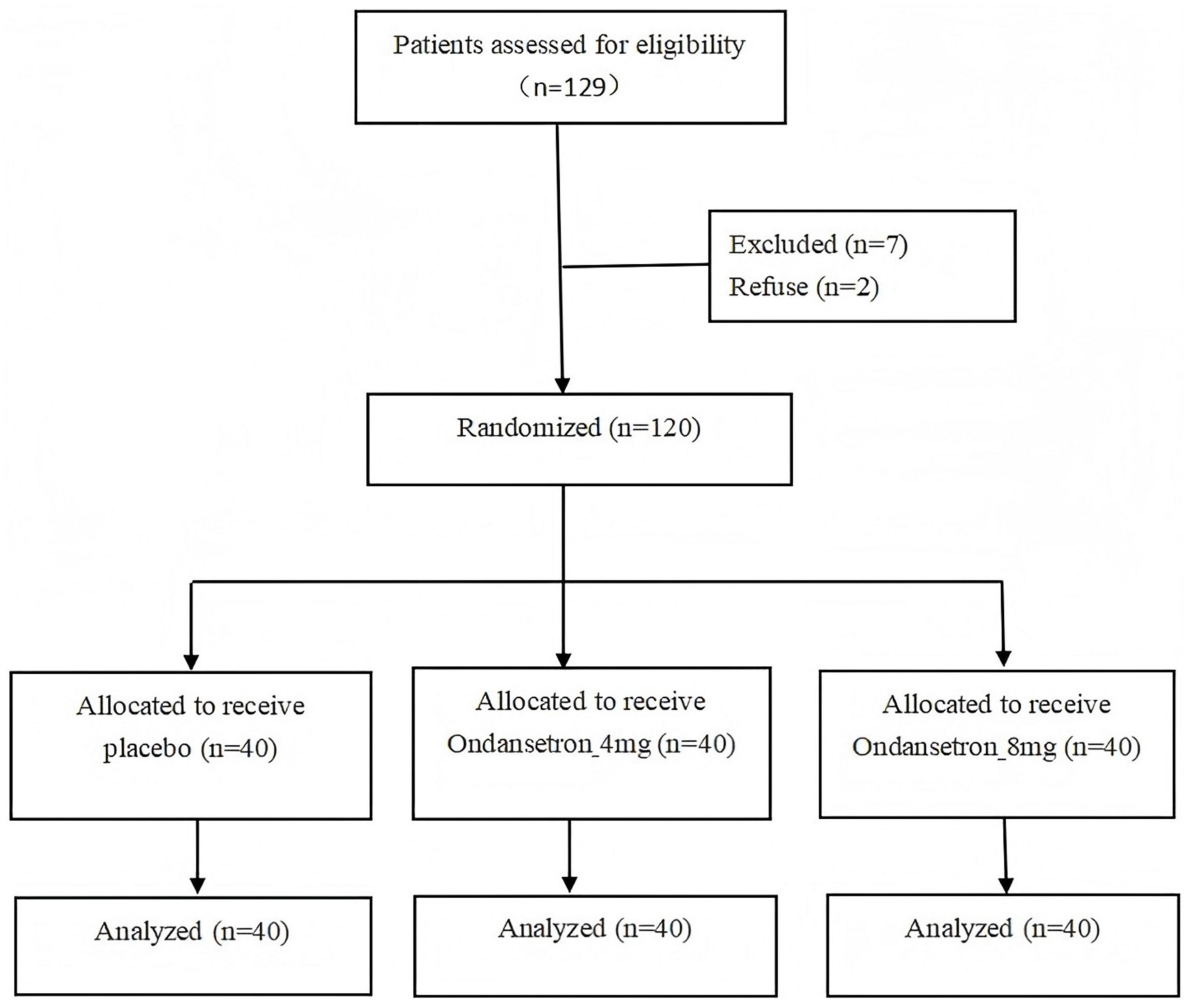
Consolidated Standards of Reporting Trials (CONSORT) flowchart showing subject allocation.

Inclusion criteria were women 18–45 years of age with primipara or multipara singleton pregnancy of at least 36 weeks duration who were scheduled for elective cesarean section under spinal anesthesia and who had an American Society of Anesthesiologists physical status score of < III. Exclusion criteria included: height < 150 cm, a body mass index score ≥ 40 kg/m^2^, gestational hypertension, eclampsia, pre-eclampsia, or chronic hypertension, a baseline systolic blood pressure of ≥160 mmHg, a hemoglobin level < 7 g/dL, fetal distress, or known fetal congenital abnormalities. For example: maternal request for cesarean section, oligohydramnios, polyhydramnios, scarred uterus, the thin uterine wall, fetal oversize, etc. The CONSORT flow diagram for this study is shown in Figure 1.

### Study procedures

One hundred and twenty patients were randomly divided into three groups of 40: a control group given normal saline (Group NS), an intervention with 4 mL of 4 mg ondansetron in normal saline (Group 4 mg), and an intervention with 4 mL of 8 mg ondansetron in normal saline (Group 8 mg). Non-invasive blood pressure, electrocardiogram, and oxyhemoglobin saturation (SpO_2_) monitoring were performed. Baseline systolic blood pressure (SBP) and heart rate (HR) were established. An indwelling 18-gauge intravenous (IV) catheter was placed in an upper limb vein for compound sodium chloride (0.85% NaCl, 0.03% KCl, and 0.033% CaCl_2_) continuous infusion and norepinephrine bolus infusion. Codes were created using a computer-generated randomization sequence and placed into serially numbered, opaque, sealed envelopes to divide the patients. Allocation concealment was double-blind. Patients received each respective treatment 5 min before spinal anesthesia. The crystalloid preload (3 mL/kg) was administered before spinal anesthesia. No fluid was administered after the initiation of anesthesia (co-load). To initiate spinal anesthesia, a 25-gauge spinal needle (Hisern Medical Device Co., Ltd., Zhejiang, China) was inserted into the L2–L3 or L3–L4 interspace in the right lateral decubitus position, and hyperbaric bupivacaine 0.5% (2.5 mL, 12.5 mg) was administered after the outflow of cerebrospinal fluid. The patients were placed in the supine position and tilted 15° to the left. Block height was assessed at T6 using the pinprick. After fetal delivery, all patients received crystalloid fluid infusion (8 mL/kg/h) until the end of surgery. SBP and HR were recorded at 60-s intervals after the administration of spinal anesthesia and every 5 min after delivery until the end of surgery.

Every 2 min, baseline SBP and HR were measured three times; prior to anesthesia, we confirmed that resting state values did not differ by more than 10%. Averages were considered baseline SBP and HR. Post-spinal anesthesia hypotension and severe hypotension were defined as SBP dropping to <80% and < 60% of baseline. If either occurred, a 6 μg i.v. bolus of norepinephrine was administered and repeated if ineffective. Hypertension was defined as SBP > 120% of baseline or > 160 mmHg. Bradycardia was defined as HR < 60 beats min^−1^ and was managed with 0.25–0.5 mg i.v. atropine. Clinical manifestations (retching or vomiting) and/or any request for antiemetics (until the end of the procedure) were considered episodes of nausea/vomiting (13).

### Primary and secondary endpoints

The performance error (PE) was used to evaluate the overall stability of SBP versus baseline and was presented through MDPE and MDAPE (14, 15). The percentage of PE (difference between each measured SBP value and baseline value), expressed as a percentage of the baseline value, was calculated for each patient. The primary outcomes were median performance error (MDPE; the median of all values of PE for each patient) and median absolute performance error (MDAPE; the median of the absolute values of PE for each patient). These presented stability of SBP within 15 min after spinal anesthesia versus baseline. Secondary outcomes included post-spinal anesthesia hypotension, severe hypotension, and adverse events (nausea or vomiting, bradycardia, hypertension, umbilical artery blood gas values, and Apgar scores).

### Sample size determination and statistical analysis

Preliminary tests (16) showed that MDPE of SBP was (median [interquartile range]:−11.89 [−17.14 to 6.48]) in patients in the NS group (without prophylactic measures). Based on this, we assume that the MDPE of SBP reduced by 6% based on-11.89% in the experimental group (prophylactic ondansetron). Using one-way analysis of variance (ANOVA) and F-test with a type I error set at 0.05 and a type II error set at 0.1 (PASS 11.0; NCSS Statistical Software, LLC, Kaysville, UT, USA), a sample size of 114 was required. Allowing for possible dropouts, 120 patients were needed, and 40 patients were allocated to each group.

The Kolmogorov–Smirnov test was used to confirm the normality of continuous variables and a one-way ANOVA was used to analyze normally distributed variables. The Kruskal–Wallis test followed by a *post hoc* Dunn test was used to analyze continuous variables that did not follow a normal distribution. ANOVA with repeated measures was used to compare SBP and HR, and a chi-square test was used to compare categorical variables. Chi-squared tests were also used for pairwise comparisons if the overall test of difference among groups was deemed statistically significant. To calculate PE-related parameters, Microsoft Office Excel (2015) was used. SPSS version 22.0 (IBM Corp., Armonk, NY, USA) was used for data analysis, and a *p*-value of <0.05 was considered significant.

## Results

Of the 129 patients eligible for inclusion in the study, 9 declined leaving 120 patients that were divided equally into 3 groups (NS group, 4 mg group, and 8 mg group). The flow chart detailing patient recruitment is shown in Figure 1. Demographic data and baseline characteristics were comparable among the three groups (Table 1).

**Table 1:** Demographic and baseline characteristics.

	NS Group (*n* = 40)	4 mg Group (*n* = 40)	8 mg Group (*n* = 40)	*p* value
Age (years)	31.05 ± 5.02	30.87 ± 4.94	30.67 ± 5.26	0.974
BMI (kg/m^2^)	28.40 ± 5.47	27.74 ± 4.37	27.74 ± 4.57	0.761
Baseline characteristics				
SBP (mmHg)	119.94 ± 10.74	120.15 ± 10.45	116.67 ± 11.09	0.145
HR (beats/min)	91.49 ± 15.3	92.77 ± 15.83	90.92 ± 11.75	0.337
Block height^a^	T6 [T4 – T6]	T6 [T5 – T6]	T6 [T5 –T6]	0.186
Time from anesthesia to delivery (min)	14.30 ± 3.11	15.90 ± 4.15	15.24 ± 3.56	0.145
Time from skin incision to delivery (min)	3.43 ± 1.88	3.9 ± 2.74	3.7 ± 1.99	0.636
Length of postoperative stay (d)	3.45 ± 0.81	3.75 ± 1.06	3.55 ± 0.81	0.321

Data are presented as mean ± SD (standard deviation) and median [interquartile range].

BMI = body mass index; SBP = systolic blood pressure; HR = heart rate; ^a^Sensory blockade was assessed using a sterile needle.

### Primary outcomes

The primary outcomes were significantly different between the three groups. (MDPE, *p* = 0.001; MDAPE, *p* = 0.002). The MDPEs were negative, indicating a bias for SBP on average to be below baseline in the three groups. The MDPEs were −12.7, −9.80, and −9.83 in the NS, 4 mg, and 8 mg groups, respectively. The magnitude of this difference was greater in the NS group than in the 4 mg and 8 mg groups (*p* = 0.003, *p* = 0.011, respectively). The MDAPEs were 13.12, 9.99, and 1.58 in the three groups, respectively. Compared to the NS group, the MDAPEs were significantly reduced in other groups with the two different doses of prophylactic ondansetron (*p* = 0.006, *p* = 0.006, respectively) (Table 2).

**Table 2:** Stability of SBP over baseline value within 15 min after post-spinal anesthesia (MDPE & MDAPE).

	NS Group (*n* = 40)	4 mg Group (*n* = 40)	8 mg Group (*n* = 40)	*p* value
MDPE (%)	−12.73 [−15.51 to −10.05]^*^	−9.80 [−12.73 to −5.35]	−9.83 [−12.69 to −7.06]	0.001
MDAPE (%)	13.12 [10.96 to 15.60]^#^	9.99 [8.07 to 12.76]	10.58 [7.40 to 12.69]	0.002

Data are presented as median [interquartile range]. MDPE = median performance error; MDAPE = median absolute performance error.

^*^p = 0.003 vs 4 mg Group; *p* = 0.011 vs 8 mg Group.

^#p = 0.006 vs 4 mg Group; p = 0.006 vs 8 mg Group.^

### Secondary outcomes

**Figure 2:**
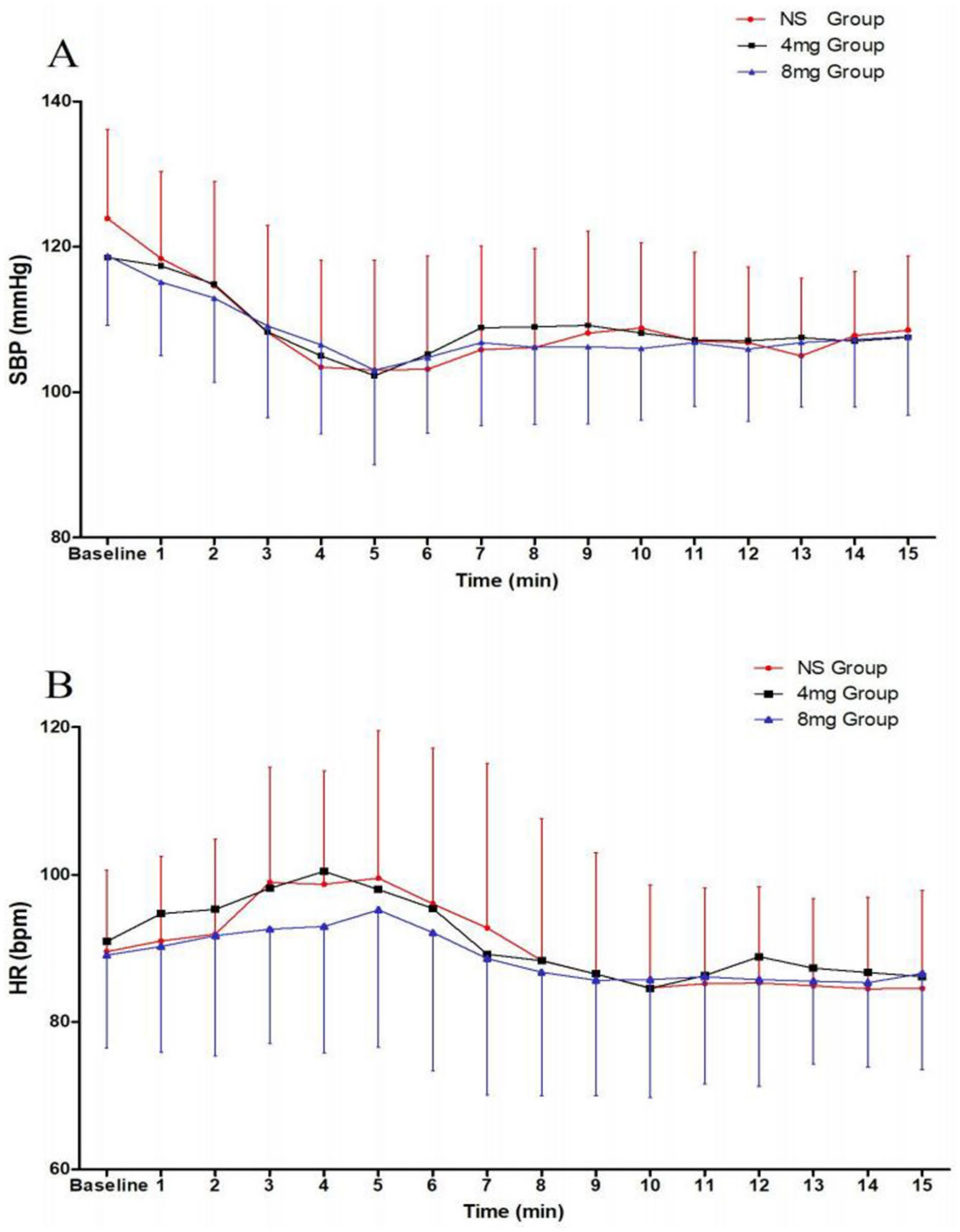
Systolic blood pressure (SBP) **(A)** and heart rate (HR) **(B)** post-spinal anesthesia. Data are shown as mean ± standard deviation.

The incidence of post-spinal anesthesia hypotension was 75, 50, and 38% in the NS, 4 mg, and 8 mg groups, respectively. There was a significant difference between the three groups (*p* = 0.002). Compared with the NS group, the incidence of post-spinal anesthesia hypotension was most decreased in the 8 mg group (*p* = 0.001). The incidence of severe post-spinal anesthesia hypotension was 8, 3, and 5% of patients in the NS, 4 mg, and 8 mg groups, respectively. The incidence of bradycardia was 7% in all three groups. The incidence of nausea and vomiting was 20, 13, and 10% in the NS, 4 mg, and 8 mg groups, respectively. No patients had hypertension in the three groups. There were no significant differences in the incidence of severe hypotension, bradycardia, nausea and vomiting, hypertension, or the doses of norepinephrine among the three groups (Table 3). There were no significant differences in umbilical artery blood gas and Apgar scores among the three groups (Table 4). SBP was higher and HR was lower in groups receiving ondansetron relative to the NS group, but no difference was observed between treatment*time among the groups (*p* = 0.152) (Figure 2).

**Table 3:** Adverse events.

	NS Group (*n* = 40)	4 mg Group (*n* = 40)	8 mg Group (*n* = 40)	*p* value
Post-spinal anesthesia hypotension, *n* (%)	30 (75)^*^	20 (50)	15 (38)	0.002
Severe post-spinal anesthesia hypotension, *n* (%)	3 (8)	1 (3)	2 (5)	0.677
Bradycardia, *n* (%)	3 (7)	3 (7)	3 (7)	0.967
Nausea and Vomiting, *n* (%)	8 (20)	5 (13)	4 (10)	0.420
Number of vasopressor boluses (ug)	6 [6–12]	6 [6–12]	6 [6–12]	0.864
Hypertension, *n* (%)	0 (0)	0 (0)	0 (0)	–

Data of adverse events are presented as number (%) and median [interquartile range].

^*^p = 0.001 vs 8 mg Group.

**Table 4:** Neonatal outcomes.

	NS Group (*n* = 40)	4 mg Group (*n* = 40)	8 mg Group (*n* = 40)	*p* value
pH	7.33 ± 0.06	7.35 ± 0.04	7.35 ± 0.05	0.111
PCO_2_ (mmHg)	43.19 ± 7.34	42.11 ± 6.62	42.88 ± 7.78	0.792
BE (mmol/L)	−2.91 ± 1.43	−2.21 ± 1.57	−2.98 ± 1.74	0.064
PO_2_ (mmHg)	23.01 ± 7.36	23.22 ± 5.98	23.26 ± 6.16	0.949
Apgar score, 1 min	9 [9–9]	9 [9–9]	9 [9–9]	0.510
< 7 at 1 min, *n* (%)	0 (0%)	0 (0%)	0 (0%)	–
Apgar score, 5 min	10 [10–10]	10 [9–10]	10 [10–10]	0.823
< 7 at 5 min, n (%)	0 (0%)	0 (0%)	0 (0%)	–

Data of adverse events and neonatal outcomes are presented as mean ± SD (standard deviation), median[interquartile range], and number (%).

PCO_2_ = partial pressure of carbon dioxide; BE = base excess; PO_2_ = partial pressure of oxygen.

## Discussion

This study revealed that prophylactic 4 mg or 8 mg ondansetron improved hemodynamic stability after spinal anesthesia in parturients undergoing elective cesarean section. There was a statistical difference in the incidence of post-spinal anesthesia hypotension among the three groups (*p* = 0.002), but there was no statistical difference between the 4 mg and 8 mg groups. Only prophylactic 8 mg ondansetron reduced post-spinal anesthesia hypotension in the cesarean section.

A meta-analysis (17) revealed suggested that ondansetron may be recommended as a prophylaxis for hypotension and bradycardia following spinal anesthesia. Sahoo et al. (18) compared the doses of norepinephrine and the decrease of mean arterial pressure (MAP) in 5 min and 6 min after spinal anesthesia in between groups, which were the preventive administration of 4 mg ondansetron (group O) and normal saline (group S), and showed that prophylactic use of 4 mg ondansetron can significantly improve the stability of maternal circulation after spinal anesthesia and reduce the need for vasopressors. In our study, the primary outcome MDAPE was significantly smaller in the 4 mg and 8 mg groups versus the NS group, indicating the maintenance of SBP on average was closer to baseline in the 4 mg and 8 mg groups. MDPE indicated a bias for SBP on average to be below baseline in all three groups, although the magnitude of this difference was greater in the NS group than in the 4 mg and 8 mg groups. The results are similar to the study by Sahoo et al. and show that prophylactic ondansetron improved maternal circulatory stability after spinal anesthesia. However, the doses of vasopressors were not statistically different in our study. The difference in the results may be due to different definitions of hypotension and inconsistent sample sizes. Sahoo et al. (18) used an SBP of <90 mmHg as the definition of hypotension and only scheduled 52 parturients. Two meta-analyses published by Gao et al. (19) and Heesen et al. (20) also concluded that ondansetron effectively reduced the incidence of post-spinal anesthesia hypotension in cesarean section. In our study, only prophylactic 8 mg ondansetron reduced the incidence of post-spinal anesthesia hypotension. Similarly, a study that explored the effect of weight-based dosing of ondansetron to reduce post-spinal anesthesia hypotension in cesarean section showed that similar dosing to our study ondansetron was not effective in reducing the incidence of hypotension in pregnant women undergoing cesarean section (11). Another study found that earlier administration of 4 mg prophylactic ondansetron contributed no benefits for lowering the dose of prophylactic phenylephrine compared to a late administration (21). This inconsistency may be related to the timing of ondansetron administration, the dose of local anesthetics used, and inconsistent sample sizes between studies.

Prophylactic ondansetron did not reduce the incidence of bradycardia after spinal anesthesia in the present study. A similar study (22) showed that 8 mg prophylactic ondansetron was more effective than normal saline in preventing post-spinal anesthesia hypotension but did not affect HR. Sudden bradycardia may be caused by the transfer of cardiac autonomic balance to the vagus nervous system or by increased baroreflex activity caused by activation of left ventricular mechanoreceptors or chemoreceptors causing the BJR. Theoretically, 5-HT3 receptor antagonists can inhibit the BJR and reduce the occurrence of bradycardia (23). This is inconsistent with our results. This might be because most bradycardia after cesarean section has been related to side effects of vasoactive drugs (phenylephrine) used to correct post-spinal anesthesia hypotension (24). This study showed that the incidence of nausea and vomiting in the 4 mg and 8 mg groups was less than in the NS group, but was not statistically significant. This is similar to a previous study in which ondansetron 8 mg has been shown to reduce the occurrence of postoperative nausea and vomiting (25). The occurrence of nausea and vomiting during cesarean section may be related to the occurrence of intraoperative hypotension, which may be related to vagus nerve excitation and brain tissue hypoxia caused by severe reduction of blood pressure. Timely correction of post-spinal anesthesia hypotension could significantly reduce the occurrence of nausea and vomiting. Besides, long-acting intrathecal opioids such as morphine are commonly administered for cesarean section and are associated with higher rates of nausea and vomiting (26).

Our study has some important limitations. First, we did not monitor invasive arterial blood pressure and invasive cardiac hemodynamic monitoring because it is not routine monitoring in healthy parturients and it is also costly. Although we measured blood pressure every minute during the first 15 min after spinal anesthesia, invasive blood pressure measurement and cardiac hemodynamic monitoring might be sufficiently sensitive to notice hemodynamic changes to promptly treat hypotension in parturients (11). In a future study, we should collect this information. Second, we did not explore the prophylactic effect of higher doses of ondansetron. Further studies are needed to determine whether higher doses of ondansetron can effectively reduce the doses of norepinephrine, bradycardia, nausea, and vomiting after spinal anesthesia in pregnant women undergoing cesarean section. The dosage of ondansetron above 0.15 mg/kg might cause umbilical arterial vasoconstriction and be harmful to the fetus (27, 28).

In conclusion, prophylactic 4 mg or 8 mg ondansetron improved hemodynamic stability after spinal anesthesia in the cesarean section, but only 8 mg reduced post-spinal anesthesia hypotension.

## Data Availability

The original contributions presented in the study are included in the article/supplementary material, further inquiries can be directed to the corresponding author.
